# Effects of Post-Deposition Annealing on ZrO_2_/n-GaN MOS Capacitors with H_2_O and O_3_ as the Oxidizers

**DOI:** 10.1186/s11671-017-2024-x

**Published:** 2017-04-11

**Authors:** Meijuan Zheng, Guozhen Zhang, Xiao Wang, Jiaxian Wan, Hao Wu, Chang Liu

**Affiliations:** Key Laboratory of Artificial Micro- and Nano-structures of Ministry of Education, and School of Physics and Technology, Wuhan University, Wuhan, 430072 China

**Keywords:** ZrO_2_, GaN, MOS, Atomic layer deposition, Post-annealing

## Abstract

GaN-based metal-oxide-semiconductor capacitors with ZrO_2_ as the dielectric layer have been prepared by atomic layer deposition. The accumulation and depletion regions can be clearly distinguished when the voltage was swept from −4 to 4 V. Post-annealing results suggested that the capacitance in accumulation region went up gradually as the annealing temperature increased from 300 to 500 °C. A minimum leakage current density of 3 × 10^−9^ A/cm^2^ at 1 V was obtained when O_3_ was used for the growth of ZrO_2_. Leakage analysis revealed that Schottky emission and Fowler-Nordheim tunneling were the main leakage mechanisms.

## Background

Gallium nitride (GaN)-based wide bandgap semiconductors have seen enormous success during the past few decades due to their intriguing properties such as high breakdown electric field (4.2 MV/cm), high saturation velocity (~3 × 10^7^ cm/s) [1], excellent chemical stability, and the ability to resist radiation damage [2]. Owing to these characteristics, GaN and its alloys can be applied in high-power electronics, such as thin film transistors and high electron-mobility transistors (HEMTs) [3, 4]. The conventional HEMTs apply Schottky barrier as the control gate which usually produces a large leakage current and, thus, results in a declined breakdown voltage and an enlarged power consumption as well as an increased noise coefficient [5, 6]. To reduce the leakage current, metal-oxide-semiconductor (MOS) structures are proposed to replace the ordinary Schottky gate [7]. However, the surface passivation of GaN is usually difficult due to the existing surface defects, dangling bonds, and some impurities, which distort the interface energy band. Hence, it is crucial to analyze and optimize the MOS structures before fabricating the MOS HEMTs.

In terms of dielectric films, high-*k* oxides are always among the candidates, because the high dielectric constant means much smaller featured size and lower power consumption [8, 9]. Many high-*k* materials such as Al_2_O_3_ [10], ZrO_2_ [11], MgO [12], and TiO_2_ [13] have been investigated for GaN-based MOS capacitors. Among them, ZrO_2_ is attractive because it has a high permittivity (~24), large bandgap (5.8 eV), and more importantly, an appropriate value of band offset with GaN (valance band offset ~1.6 eV, conduction band offset ~1.1 eV) [14]. As for the deposition methods, atomic layer deposition (ALD) has become the mainstream technique due to its unique self-limited reactions, which demonstrates many advantages such as precise thickness control, high uniformity over a large area, and excellent conformity in many complex nanostructures. In 2013, P. von Hauff et al. reported a kind of GaN-based MOS capacitors with ZrO_2_ as the dielectric layer. The capacitance reached 3.8 μF/cm^2^ in the accumulation region. However, the leakage current reached an enormous 0.88 A/cm^2^ at 1 V which was too large to be applied in electronic devices [15]. In 2014, Gand Ye et al. investigated the band alignment of ZrO_2_ and GaN using X-ray photoelectron spectroscopy [16]. However, the related devices were not fabricated.

In this work, we systematically studied the properties of ZrO_2_ films grown on n-GaN substrates by ALD. H_2_O or O_3_ was used as the oxidizer to examine which precursor was more effective to grow high-quality ZrO_2_ films. In addition, post-annealing treatments were carried out to improve the electrical performances of the MOS capacitors. Meanwhile, the leakage mechanisms of the MOS capacitors were also discussed.

## Methods

**Fig. 1:**
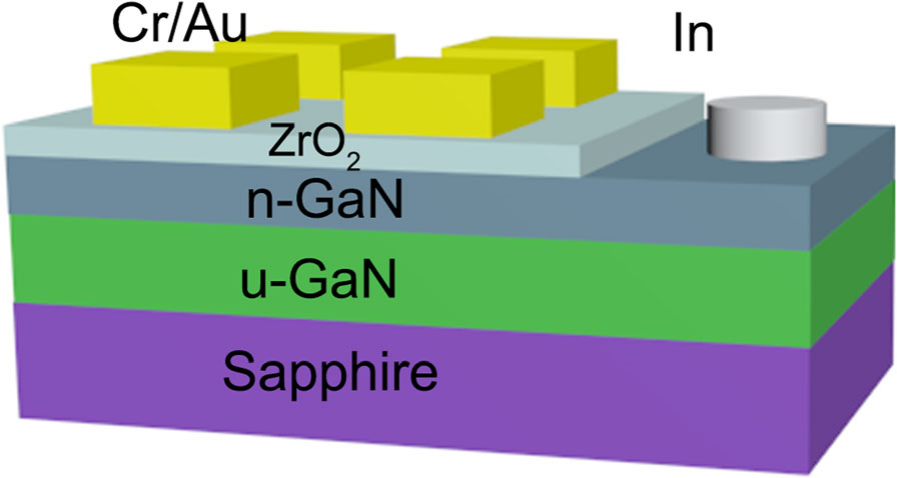
The schematic diagram of Cr/Au/ZrO_2_/n-GaN MOS capacitors

Commercially available n-type GaN substrates were purchased from Huacan Semitek. A 3-μm-thick Si-doped n-type GaN layer with a carrier concentration of 2 × 10^18^/cm^3^ was grown on c-plane sapphire substrate by metal organic chemical vapor deposition with an un-doped GaN buffer layer. Firstly, the n-GaN substrates were ultrasonically cleaned in acetone for 10 min, followed in ethanol also for 10 min. The samples were purged by high-pressure N_2_ gas to remove any remaining particles on the surface. Then, n-GaN substrates were transferred into ALD (Beneq TFS-200) chamber, and ZrO_2_ films with a thickness of 20 nm were deposited at 200 °C. Tetrakis-dimethylamino zirconium (TDMAZ) was used as the Zr precursor which was heated to 85 °C to produce enough vapor pressure. Water or ozone was used as the oxidizer. H_2_O was heated to 40 °C, and O_3_ was produced by an ozone generator. For H_2_O oxidant, the growth rate was about 0.885 Å/cycle, and 226 cycles were performed to grow 20 nm thick ZrO_2_. For O_3_ oxidant, the growth rate was about 0.95 Å/cycle and a total of 210 cycles was needed for the same thickness. The top isolated Cr/Au (20/50 nm) electrodes were formed on ZrO_2_ films by using an ordinary lift-off process. Figure 1 shows the device diagram of the GaN-based MOS capacitors. In order to improve the ZrO_2_ films and the interfacial qualities, a post rapid thermal annealing (RTA) process was performed in a temperature range from 300 to 600 °C in N_2_ atmosphere. A spectroscopic ellipsometer (J. A. Woollam alpha-SE) system was used to measure the ZrO_2_ film thickness. The crystallization and interface of MOS capacitors were evaluated by high-resolution transmission electron microscopy (HRTEM, JEOL JEM2010 FEF UHR). The capacitance density versus voltage (*C*-*V*) and current density versus voltage (*I*-*V*) characteristics were measured at room temperature by using a Keithley 4200 semiconductor analyzer.

## Results and Discussion

**Fig. 2:**
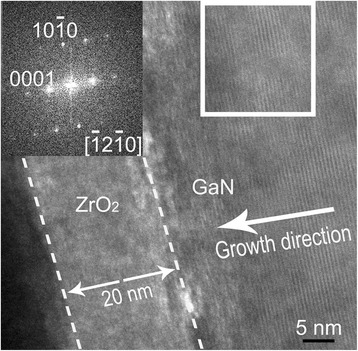
Cross-sectional HRTEM image of MOS capacitors with O_3_ as the oxidizer (the *inset* is the related FFT image)

Figure 2 shows the cross-sectional HRTEM image of MOS capacitors with O_3_ as the oxidizer. As is shown, the interface is distinct and the thickness of the ZrO_2_ films is about 20 nm. Obviously, the ZrO_2_ films exhibit an amorphous phase. Besides, the growth direction of GaN is along [0001], deduced from the corresponding FFT image (shown in the inset of HRTEM image).

**Fig. 3:**
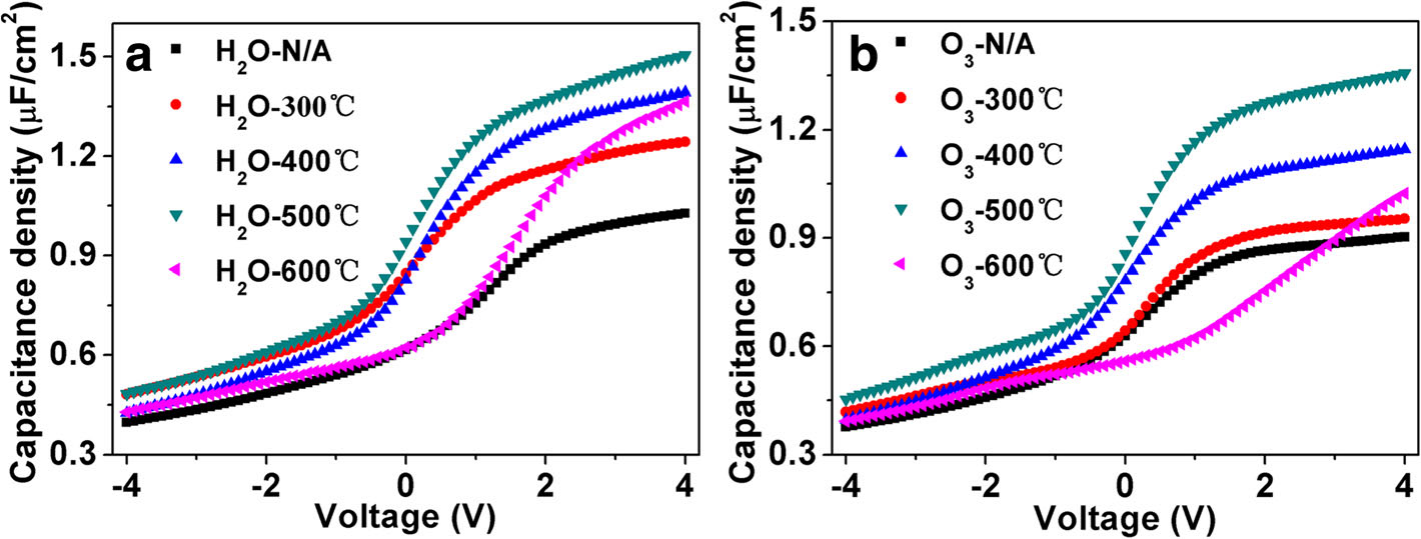
*C*-*V* characteristics of MOS capacitors under different annealing temperatures. **a** H_2_O as the oxidizer. **b** O_3_ as the oxidizer

**Fig. 4:**
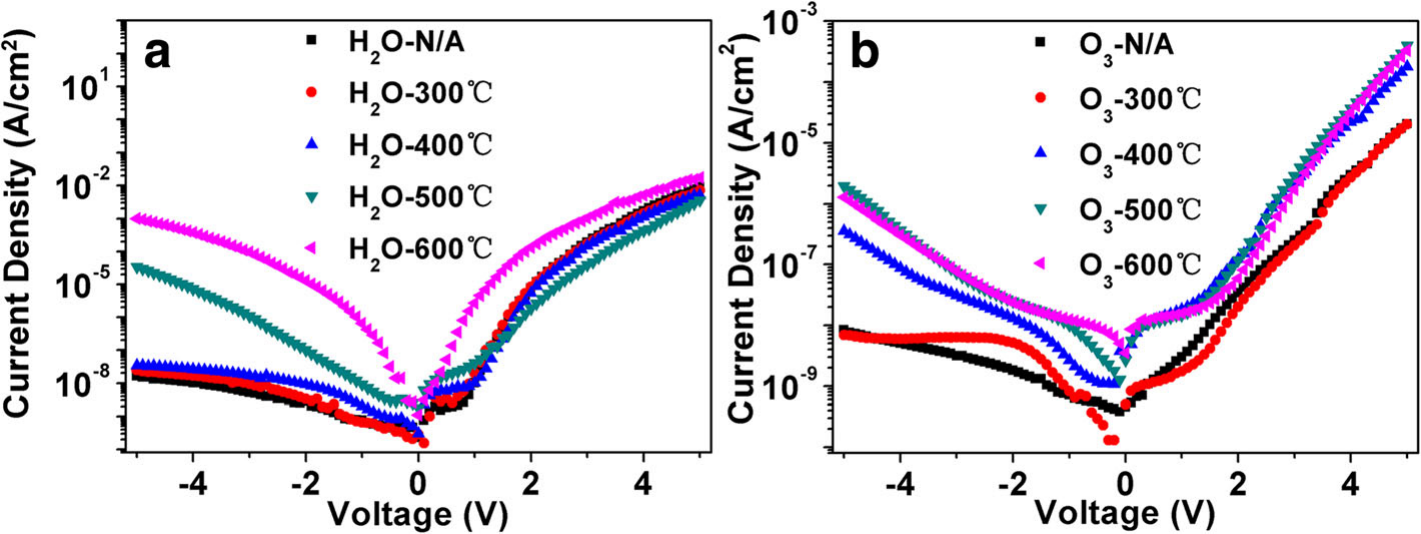
*I*-*V* characteristics of MOS capacitors under different annealing temperatures. **a** H_2_O as the oxidizer. **b** O_3_ as the oxidizer

**Fig. 5:**
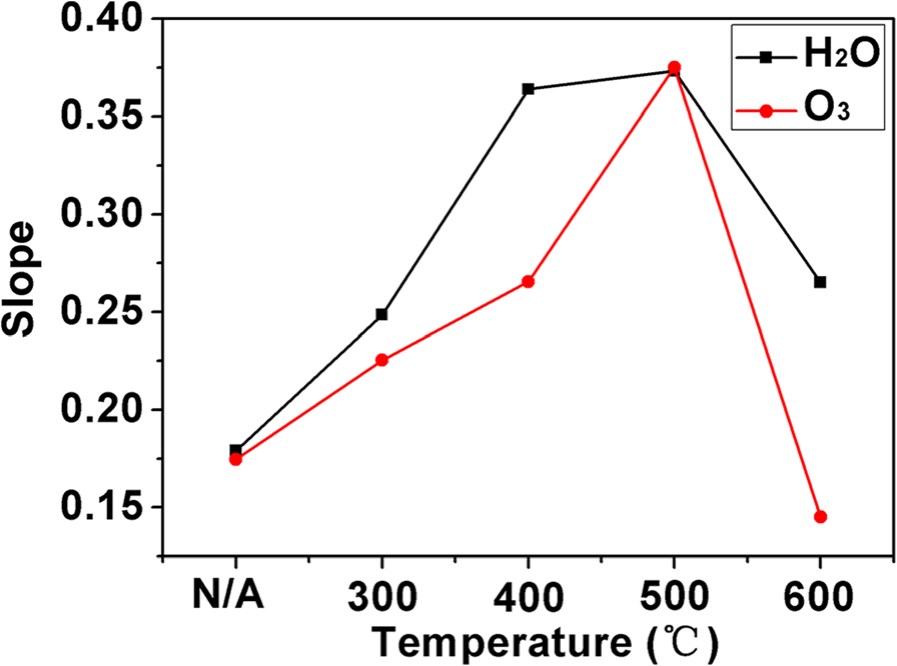
The maximum ratio of capacitance density and voltage of the capacitors with different annealing temperatures

Figure 3 shows the *C*-*V* characteristics of ZrO_2_/GaN structure measured at 10 kHz with different annealing temperatures. A depletion region (low capacitance state) in the negative voltage range and an accumulation region (high capacitance state) in the positive voltage range were clearly observed for MOS capacitors with either H_2_O or O_3_ as oxidizer. The capacitance density went up gradually as the annealing temperature increased, which can be attributed to the improved crystalline quality of ZrO_2_ films [17]. However, the MOS capacitors with H_2_O as the oxidizer showed a higher capacitance than that with O_3_ oxidant. The reason was probably due to that the former have more oxygen vacancies and hydroxyl residuals [18]. These defects and impurities, on the one hand, can provide extra inherent electric dipoles which contributed to a part of capacitance. On the other hand, a larger leakage current may be produced, as seen from Fig. 4. For H_2_O as the oxidizer, the capacitance density was enhanced from the initial 1.24 to 1.50 μF/cm^2^ when the annealing temperature reached at 500 °C. This is a better inclination because the low and high capacitance states became much more distinguishable, and thus, the switching speed which can be described by the slopes of *C*-*V* curves from low to high states increased gradually, as shown in Fig. 5. For O_3_ as the oxidizer, the variation tendency was similar to that of H_2_O oxidizer. The capacitance increased from 0.95 to 1.36 μF/cm^2^ when the device was annealed at 500 °C. The slope also reached to the maximum at 500 °C, as seen from Fig. 5. However, the capacitance began to decline when the annealing temperature reached at 600 °C, which can be ascribed to the formation of interfacial layers such as GaO_*x*_ [19].

**Fig. 6:**
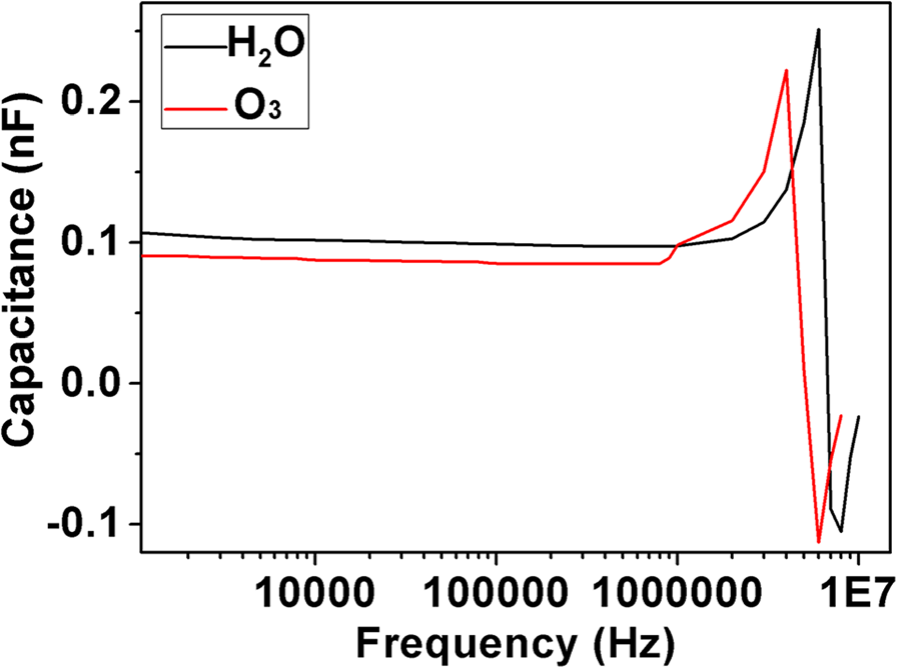
C-F plot of as-deposited films with H_2_O or O_3_ as the oxidizer

Figure 6 shows the C-F features of the capacitors under a DC bias of 4 V. Both capacitors can work normally in a frequency range from 1 kHz to 1 MHz. An abrupt increase of the capacitance was observed for both capacitors with H_2_O and O_3_ as oxidizers, which can be attributed to the resonance effect at high frequencies.

In terms of leakage properties, as shown in Fig. 4, the leakage current in the negative voltage range was remarkably lower than that in the positive voltage range for capacitors with H_2_O or O_3_ as the oxidizer. This phenomenon can be attributed to the formation of the depletion region when the negative voltage was added. The depletion region decreases the electric field intensity on the dielectric layer, which resulted in a much lower leakage current density. The leakage current density of the capacitors without annealing was improved from 1 × 10^−8^ to 3 × 10^−9^ A/cm^2^ at 1 V when O_3_ was used as the oxidizer instead of H_2_O. This could be explained in this way that O_3_ is highly volatile and has a stronger oxidizing ability than H_2_O, based on the fact that the prepared ZrO_2_ films using O_3_ oxidizer have less impurities [18] and more accurate stoichiometry composition. The improved ZrO_2_ qualities enhanced the insulating properties. As the annealing temperature gradually increased from 300 to 600 °C, the leakage current increased correspondingly because of the increased crystalline boundaries which served as the leakage channels.

**Fig. 7:**
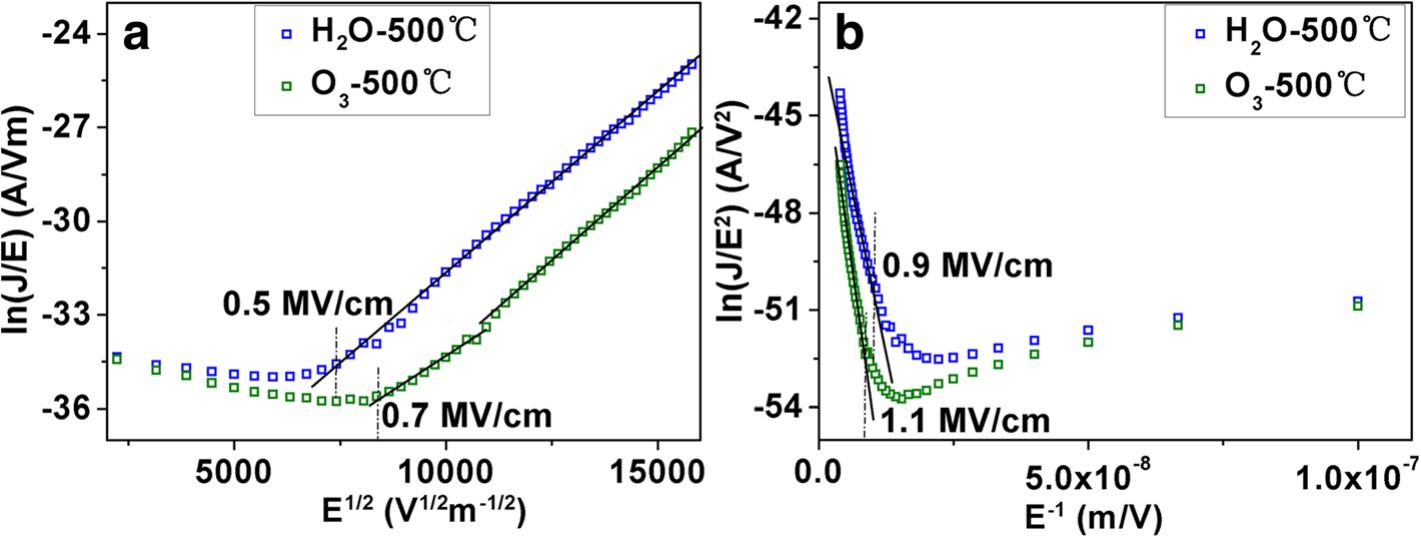
The conductive mechanism of MOS capacitors annealed at 500 °C. **a** Schottky emission. **b** F-N tunneling

It is important to understand the leakage mechanism because it helps us to find out effective ways to reduce the leakage current. Here, Schottky emission and Fowler-Nordheim (F-N) tunneling models [20] are used to analyze the *I*-*V* curves. Schottky emission is primarily applied in low or moderate electric fields. The relationship of ln(J/E) vs E^1/2^ should be linear if the leakage conduction follows the Schottky emission. F-N tunneling is mainly applied to describe the leakage current in high electric fields. The plot of ln(J/E^2^) vs E^−1^ should be a straight line if the F-N tunneling exists. Note the fact that the maximum capacitance density was obtained at the annealing temperature of 500 °C; the leakage current for this case was studied in detail in the following. For capacitors with H_2_O oxidant, as shown in Fig. 7a, the plot of ln(J/E) vs E^1/2^ presented a straight line when the electric field was over 0.5 MV/cm. However, the Schottky emission occurred when the electric field exceeded 0.7 MV/cm for capacitors with O_3_ oxidant. This indicated that ZrO_2_ films grown with O_3_ have a higher Schottky barrier than that with H_2_O oxidant. The increased Schottky barrier height was mostly due to less impurities and defects. Figure 7b shows the ln(J/E^2^) versus E^−1^ plots of the capacitors. It was found that F-N tunneling dominated when the electric field exceeded 0.9 MV/cm for the capacitors with H_2_O as the oxidant. For capacitors with O_3_ oxidant, however, this bar increased to 1.1 MV/cm, above which the F-N tunneling dominated. This demonstrated again that ZrO_2_ films with O_3_ as the oxidant have better performance.

## Conclusions

In summary, we have fabricated GaN-based MOS capacitors with ZrO_2_ as dielectrics. We observed clearly distinguished accumulation and depletion regions from *C*-*V* results. The accumulated capacitance increased gradually as the annealing temperature increased. In addition, a low leakage current density of 3 × 10^−9^ A/cm^2^ at 1 V was obtained by using O_3_ oxidant. Based on the leakage current analyses, it can be concluded that Schottky emission dominated at low fields, while F-N tunneling governed at high fields.

## References

[1] Ozbek AM, Baliga BJ (2011). Planar nearly ideal edge-termination technique for GaN devices. IEEE Electron Device Lett.

[2] Kim HY, Anderson T, Mastro MA, Freitas JA, Jang S, Hite J, Eddy CR, Kim J (2011). Optical and electrical characterization of AlGaN/GaN high electron mobility transistors irradiated with 5 MeV protons. J Cryst Growth.

[3] Zimmermann T, Deen D, Cao Y, Simon J, Fay P, Jena D, Xing HG (2008). AlN/GaN insulated-gate HEMTs with 2.3 A/mm output current and 480 mS/mm transconductance. IEEE Electron Device Lett.

[4] Huang W, Chow TP, Niiyama Y, Nomura T, Yoshida S (2009). Experimental demonstration of novel high-voltage epilayer RESURF GaN MOSFET. IEEE Electron Device Lett.

[5] Dora Y, Han S, Klenov D, Hansen PJ, No KS, Mishra UK, Stemmer S, Speck JS (2006). ZrO_2_ gate dielectrics produced by ultraviolet ozone oxidation for GaN and AlGaN/GaN transistors. J Vac Sci Technol B.

[6] Liao CN, Zou XM, Huang CW, Wang JL, Zhang K, Kong YC, Chen TS, Wu WW, Xiao XH, Jiang CZ, Liao L (2015). Low interface trap densities and enhanced performance of AlGaN/GaN MOS high-electron mobility transistors using thermal oxidized Y_2_O_3_ interlayer. IEEE Electron Device.

[7] Chung JW, Roberts JC, Piner EL, Palacios T (2008). Effect of gate leakage in the subthreshold characteristics of AlGaN/GaN HEMTs. IEEE Electron Device Lett.

[8] Xie Q, Deng S, Schaekers M, Lin D, Caymax M, Delabie A, Qu XP, Jiang YL, Deduytsche D, Detavernier C (2012). Germanium surface passivation and atomic layer deposition of high-k dielectrics—a tutorial review on Ge-based MOS capacitors. Semicond Sci Tech.

[9] Suzuki M (2012). Comprehensive study of lanthanum aluminate high-dielectric-constant gate oxides for advanced CMOS devices. Materials.

[10] Yang S, Tang ZK, Wong KY, Lin YS, Liu C, Lu YY, Huang S, Chen KJ (2013). High-quality interface in MIS structures with in situ pre-gate plasma nitridation. IEEE Electron Device Lett.

[11] Bothe KM, von Hauff PA, Afshar A, Foroughi-Abari A, Cadien KC, Barlage DW (2013). Electrical comparison of and gate dielectrics on GaN. IEEE T Electron Dev.

[12] Kim J, Gila B, Mehandru R, Johnson JW, Shin JH, Lee KP, Luo B, Onstine A, Abernathy CR, Pearton SJ, Ren F (2002). Electrical characterization of GaN metal oxide semiconductor diodes using MgO as the gate oxide. J Electrochem Soc.

[13] Chou BY, Lee CS, Yang CL, Hsu WC, Liu HY, Sun WC, Wei SY, Yu SM (2014). TiO_2_-dielectric AlGaN/GaN/Si metal-oxide-semiconductor high electron mobility transistors by using nonvacuum ultrasonic spray pyrolysis deposition. IEEE Electron Device Lett.

[14] Robertson J, Falabretti B (2006). Band offsets of high K gate oxides on III-V semiconductors. J Appl Phys.

[15] von Hauff P, Afshar A, Foroughi-Abari A, Bothe K, Cadien K, Barlage D (2013). ZrO_2_ on GaN metal oxide semiconductor capacitors via plasma assisted atomic layer deposition. Appl Phys Lett.

[16] Ye G, Wang H, Arulkumaran S, Ng GI, Li Y, Liu ZH, Ang KS (2014). Band alignment between GaN and ZrO_2_ formed by atomic layer deposition. Appl Phys Lett.

[17] Weinreich W, Wilde L, Müller J, Sundqviet J, Erben E, Heimann J, Lemberger M, Bauer AJ (2013). Structural properties of as deposited and annealed ZrO_2_ influenced by atomic layer deposition, substrate, and doping. J Vac Sci Technol A.

[18] Zhang GZ, Wu H, Chen C, Wang T, Wang PY, Mai LQ, Yue J, Liu C (2014). Transparent capacitors based on nanolaminate Al_2_O_3_/TiO_2_/Al_2_O_3_ with H_2_O and O_3_ as oxidizers. Appl Phys Lett.

[19] Ye G, Wang H, Ng SLG, Ji R, Arulkumaran S, Ng GI, Li Y, Liu ZH, Ang KS (2014). Influence of post-deposition annealing on interfacial properties between GaN and ZrO_2_ grown by atomic layer deposition. Appl Phys Lett.

[20] Zhang GZ, Wu H, Chen C, Wang T, Wu WH, Yue J, Liu C (2015). Transparent nanotubular capacitors based on transplanted anodic aluminum oxide templates. ACS Appl Mater Inter.

